# Kartogenin Improves Osteogenesis of Bone Marrow Mesenchymal Stem Cells via Autophagy

**DOI:** 10.1155/2022/1278921

**Published:** 2022-12-22

**Authors:** Huichun Yan, Tingting Yu, Jing Li, Ting Zhang, Qian Li, Yanheng Zhou, Dawei Liu

**Affiliations:** ^1^Department of Orthodontics, Peking University School and Hospital of Stomatology, Beijing 100081, China; ^2^National Clinical Research Center for Oral Diseases & National Engineering Laboratory for Digital and Material Technology of Stomatology, Beijing 100081, China; ^3^Beijing Key Laboratory of Digital Stomatology, Beijing 100081, China; ^4^State Key Laboratory of Military Stomatology, Xi'an 710000, China

## Abstract

Kartogenin (KGN), a novel small-molecule compound, has been considered a promising chondrogenic promoter in cartilage regeneration. However, whether KGN also participates in osteogenesis and bone regeneration remains unclear. This research was designed to explore the roles of KGN on osteogenic differentiation in bone marrow mesenchymal stem cells (BMMSCs) as well as determine the possible mechanism of osteogenesis. We revealed that KGN enhanced the osteogenic differentiation capacity of BMMSCs without affecting cell proliferation, during which autophagic activities and the expression of autophagy-related genes were promoted. Moreover, KGN upregulated the phosphorylation level of the Smad1/5/9 signaling, and inhibition and activation of Smad signaling were also applied to validate the involvement of Smad in BMMSCs during KGN treatment. In summary, this study shows that KGN promotes osteogenic differentiation of BMMSCs through enhancing autophagic levels and upregulating Smad1/5/9 signaling mechanically.

## 1. Introduction

Bone defects can occur due to various reasons, such as trauma, infections, and congenital malformations. Although bone tissue has the capacity for self-healing, many bone defects or fractures remain a major challenge in regeneration medicine owing to certain disease states or the defect size. Research on bone marrow mesenchymal stem cells (BMMSCs) has provided new treatment strategies for complex bone defect diseases [1, 2]. However, most of the current therapies for bone defect repair using BMMSCs suffer from weaknesses such as poor osteogenic differentiation ratio, low level of bone fusion, and unsatisfied osteogenesis stability; therefore, there is an urgent need for further exploration of the BMMSC differentiation mechanism and optimization of their directional differentiation regulation in bone tissue engineering to promote stem cell-based bone defect repair and reconstruction. Kartogenin (KGN) is a novel small molecular compound which was studied by Johnson et al. [3] first in 2012. It was demonstrated to be capable of exerting chondroprotective effects in vitro by inducing chondrogenic differentiation of mesenchymal stem cells (MSCs) and proved efficacious in osteoarthritis animal models. Benefitting from the high stability, KGN has been broadly explored for tissue regeneration, especially for cartilage repair. To date, it has been reported that KGN can induce the differentiation of MSCs into chondrocytes both in vivo and vitro [4, 5]. Moreover, it has been studied in the fields of tendon-bone healing, wound healing, and limb development [6]. Nevertheless, there is a relative paucity of research on the effects of KGN on bone regeneration. According to Wang et al. [7], KGN promoted intracellular antioxidant activity by activating AMPK-SIRT1 signaling pathways, thereby promoting osteogenesis. Therefore, it is interesting to determine whether KGN can promote the osteogenic activity of BMMSCs and the specific mechanisms of KGN in stem cells.

MSCs have a multidifferentiation capacity and are able to maintain bone homeostasis, which has been broadly studied in the field of bone regeneration [8, 9]. Decker al. [10] revealed that KGN activates transforming growth factor-*β*/Drosophila mothers against decapentaplegic protein (TGF-*β*/Smad) signaling pathway, upregulates the phosphorylation level of Smad2/3, and stimulates mouse embryonic limb development. As an important signaling pathway, TGF-*β*/Smad is indispensable for mediating the osteogenic differentiation commitment of BMMSCs [11, 12]. Phosphorylation of Smad1/5/9 mediated by the bone morphogenetic protein- (BMP-) 2 receptor causes activated isoforms to associate with Smad4, followed by their translocation into cell nucleus, inducing osteogenesis-related gene transcription, making Smad1/5/9 signaling essential for MSC osteogenic signaling [12]. Comprising a subset of TGF-*β* superfamily, BMPs modulate numerous biological processes, such as formation of bone and cartilage during embryogenesis [13], and can result in Smad-specific transcription factor phosphorylation [14]. Among the Smad proteins, Smad1, Smad5, and Smad9 are closely associated with osteogenic differentiation [15]. Following the binding of BMPs with the specific receptor, phosphorylation of BMP-responsive Smad 1, 5, and 9 results in complex formation of R-Smads, accumulating with the common-mediator Smad4 in the nucleus, which is dedicated to osteogenesis-related gene expression regulation [16, 17], such as the transcription of Runt-related transcription factor 2 (Runx2), an essential regulatory factor in osteogenesis, leading to the initiation of the expression of other osteogenic genes [12, 18]. On this basis, whether KGN can upregulate the expression of Smad1/5/9 to promote BMMSC osteogenic differentiation needs to be confirmed.

Autophagy is a highly conserved fundamental cellular biological process that helps maintain homeostasis and enhances cell survival under cellular stress conditions [19]. Autophagy is also indispensable in cell trilineage differentiation [20], such as osteogenic differentiation [21]. In recent years, there have been studies concentrating on autophagy and osteogenic effects of MSCs. It has been reported that autophagy can enhance osteogenesis by mediating osteoblast differentiation and mineralization, especially affecting autophagosomes in extracellular calcium transportation [22]. Autophagic activity is also closely concerned with MSC differentiation potential by regulating either adipogenic or osteoblastic lineages, and autophagic level could influence bone-related cell survival rate under unfavorable situations [19]. Several markers have been proposed for monitoring autophagy, such as LC3, as well as autophagy-related genes such as LC3, Atg7, and Beclin1. LC3 generally participates in the elongation-closure of autophagosome formation and is present in two forms [23]: LC3-I, which is distributed in the cytoplasm and unconjugated to lipids, and LC3-II, located within the autophagosome membrane and conjugated to the lipid phosphatidylethanolamine, is widely used as a specific autophagosome marker to access autophagic flux.

The purpose of this research was to investigate the effects of KGN in promoting MSC osteogenesis and explore the possible mechanisms. Our findings are expected to provide new insights into small molecular compound-induced BMMSC osteogenesis in bone tissue engineering as well as new direction for clinical complex bone defect repair and reconstruction.

## 2. Materials and Methods

### 2.1. Isolation and Culture of BMMSCs

C57BL/6J mice (6-8 wk; female) were purchased from SiPeiFu Biotechnology Co., Ltd. (SPF; Beijing, China). All nucleated cells were obtained from mouse femurs and tibia bone marrow, and cells (1.5 × 10^7^ cells per 100 mm culture dish) (Corning, NY, USA) were seeded at 37°C, 5% CO_2_. After 2 d, nonattached cells were discarded, and adherent cells were cultured for 14 d in complete alpha minimum essential medium (Biological Industries, Beit Haemek, Israel) adding fetal bovine serum (20%; Gibco), L-glutamine (2 mM; Gibco), 2-mercaptoethanol (55 *μ*M; Gibco), and penicillin/streptomycin (1%; Gibco). The isolation and identification of MSCs were described in our previous studies [24–26]. The medium was refreshed per three days, and then, colony-forming attached cells were passaged once for further experimental use.

### 2.2. Cell Proliferation Assay

The survival rates of BMMSCs were performed using Cell Counting Kit-8 (CCK-8) assay (Solarbio). BMMSCs were cultured with medium (100 *μ*L per well) in the 96-well plates at a density of 1.0 × 10^3^ per well. CCK-8 reagent was diluted with culture medium to prepare the working solution after 24 h cultivation and incubated for extra 1.5 h as the instructions suggested. Subsequently, optical density value measurement was performed by a microplate reader (Bio-Rad, Virginia, USA) at the 450 nm wavelength. We performed this assay at 0 h, 12 h, and 24 h. Proliferation of the MSC population was also examined using a Ki67 incorporation assay. Mouse BMMSCs were seeded and cultured on *φ*20 mm glass chamber slide (Corning). The cultures were then incubated with medium containing Ki67 solution (1 : 100; Invitrogen, CA, USA) for another 20 h and stained using a Ki67 Staining Kit (Invitrogen). The positive Ki67 cell and overall cell counts were taken, respectively, and the proportions were calculated.

### 2.3. Transmission Electron Microscopy

Transmission electron microscope (TEM) was applied to observe autophagosomes. Cells were fixed in 4% formaldehyde and washed in PBS, followed by postfixation in 1% OsO_4_ at 4°C overnight. The samples were then dehydrated stepwisely in ascending ethanol, washed with propylene oxide, and then embedded with epoxy resin as previous study described [27]. Sections were stained with lead citrate as well as uranyl acetate.

### 2.4. Osteogenic Differentiation Assay

BMMSCs at passage 2 were prepared for osteogenic induction, and the osteogenic medium was used for BMMSC culture which contained complete culture medium plus ascorbic acid (50 mg/L; Sigma-Aldrich, MO, USA), *β*-glycerophosphate (10 mM; Sigma-Aldrich), and dexamethasone (10 nM; Sigma-Aldrich), with fresh OM replaced every three days. After two weeks of induction, 4% paraformaldehyde (Solarbio, Beijing, China) was used for cell fixing. The formation of mineralized nodule was detected using Alizarin Red Staining (ARS) kit (Sigma-Aldrich). Quantification analysis of positively staining areas was performed by Image Pro Plus 6.0v (Media Cybernetics, CA, USA) and presented by ratio.

### 2.5. Western Blotting Analysis

Total proteins of BMMSCs were obtained by RIPA buffer (Solarbio) adding the inhibitor cocktail of protease/phosphatase (Thermo Fisher Scientific, MA, USA). BCA protein assay kit (Beyotime, Shanghai, China) was used for concentration quantification. Equal amounts of protein suspension were applied on a 4-12% SDS-polyacrylamide gel (Beyotime) and then electroblotted onto polyvinylidene difluoride membrane (Thermo). Membranes were then blocked with 5% nonfat milk in TBST. Primary antibodies mouse-monoclonal anti-Runx2 (1 : 1,000; Santa Cruz, CA, USA; sc-101145), mouse-monoclonal anti-alkaline phosphatase (Alp) (1 : 1,000; Santa Cruz, CA, USA; sc-365765), rabbit-polyclonal anti-autophagy-related gene 7 (Atg7; 1 : 1,000; Proteintech, NJ, USA; 10088-2-AP), rabbit-polyclonal anti-microtubule-associated protein light chain 3 (LC3; 1 : 1,000; Cell Signaling Technology, MA, USA; 4108S), rabbit-polyclonal anti-Smad1/5/9 (1 : 1,000; Affinity Biosciences, CA, USA; AF0614), and rabbit-polyclonal anti-phosphorylated-Smad1/5/9 (pSmad1/5/9; 1 : 1,000; Affinity Biosciences; AF8313) were performed overnight in order to the membranes' molecular probing. Then, membrane incubation was implemented using horseradish peroxidase-conjugated mouse or rabbit secondary antibodies (1 : 15,000; Beyotime), after which SuperSignal West Pico Substrate (Thermo) was applied for band enhancement. Quantification analysis was conducted by ImageJ 16.0v software and relatively normalized to *β*-actin (1 : 5,000; Zhongshan Golden Bridge Biotechnology, China).

### 2.6. Immunofluorescent Staining

BMMSCs were harvested and treated with 4% paraformaldehyde and 0.1% Triton X-100 solution (Beyotime) for fixation and permeabilization, respectively, and afterwards incubated with 5% goat serum (Beyotime). Then BMMSCSs were probed with primary antibodies against LC3, Runx2, and ALP (1 : 100) overnight at 4 degree Celsius, and the secondary antibody (1 : 200, Beyotime) was then used for another an hour at room temperature. DAPI (Beyotime) was used for nucleuses counterstaining. The laser scanning confocal microscope was employed for staining visualization (LSM 510; Zeiss, Germany). The Image Pro Plus 6.0v software was performed for quantitative analytical purposes.

### 2.7. Quantitative Reverse Transcription-Polymerase Chain Reaction (qRT-PCR)

Total cellular RNA extraction was firstly performed with TRIzol reagent (Invitrogen), and then, complementary DNA was synthesised with a PrimeScript™ RT reagent Kit (TaKaRa, Tokyo, Japan) and gene-specific primers (Table 1). qRT-PCR was conducted by SYBR Green Q-PCR Master Mix (Thermo) on 7900HT Fast-Time PCR System. Relative expression levels were analyzed using 2−*ΔΔ*Ct calculating method with normalization to GAPDH.

### 2.8. Reagents and Chemicals

KGN (Selleck Chemicals, TX, USA) was dissolved with dimethylsulfoxide (DMSO) as the stock solution. Cells treated with 0.005% DMSO were used as controls. Other reagents applied in the study are listed as follows: 3-methyladenine (3-MA; Selleck Chemicals, TX, USA) (5 mM for 24 h) was used as autophagy inhibitor. Both the signal inhibitor LDN-193189 (LDN; 0.5 *μ*M for 12 h) and the activator SB4 (0.1 *μ*M for 24 h) were purchased from MedChemExpress (MCE, NJ, USA).

### 2.9. Statistical Analysis

Data processing and analysis were conducted with SPSS 23.0 v (IBM, NY, USA) as well as GraphPad Prism 8.1v for Mac (GraphPad Software, CA, USA). All values calculated are shown as the mean and standard deviation (mean ± S.D.), representing at least 3 independent experiments. Comparison of two groups was executed by independent sample *t* test analysis or Mann–Whitney rank-sum test. For difference analysis among more than two groups, one-way ANOVA with Tukey's test was conducted. Criteria for statistical significance were set at *p* < 0.05.

## 3. Results

### 3.1. KGN Promotes the Osteogenic Capacity of BMMSCs

**Figure 1:**
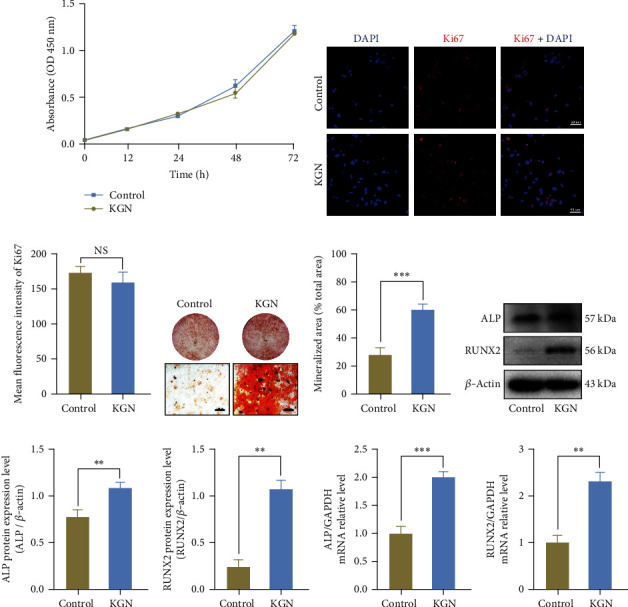
KGN enhances the osteogenic ability of BMMSCs without affecting cell proliferation. Cell proliferation capacity of BMMSCs was measured by CCK-8 (a), immunofluorescent staining (b) of Ki67, and semiquantification (c) of (b) between the control and KGN-treated BMMSCs. *p* > 0.05, independent Student's *t* test. ARS staining detection (d) and semiquantification (e) of mineralized nodules. KGN promoted osteogenesis of BMMSCs. Scale bar = 500 *μ*m. ^∗∗∗^*p* < 0.001, independent Student's *t* test. Western blotting (f) and semiquantification analysis (g, h) and qRT-PCR (i, j) of osteogenic markers ALP and Runx2. KGN upregulated the expression of osteogenic genes. ^∗∗^*p* < 0.01 and ^∗∗∗^*p* < 0.001, independent Student's *t* test.

The concentration of KGN has been suggested in previous studies [28–32], and the optimal concentration of KGN for osteogenesis in BMMSCs was 10 *μ*M in this study (Figure S1). To determine whether proliferation capacity was affected by KGN, CCK-8 was performed, and the result suggested that single KGN treatment of 10 *μ*M for 72 h did not influence cell growth (Figure 1(a)). Ki67, a marker of cell proliferation activity [33], was also measured by immunofluorescence staining, and it showed no statistical difference (Figures 1(b) and 1(c)). Next, we tested whether KGN promotes the osteogenic ability of BMMSCs. ARS staining demonstrated that matrix mineralization was visibly enhanced by KGN treatment for 14 days compared with the control group (Figures 1(d) and 1(e)). Western blotting analysis also indicated that KGN upregulated ALP protein levels in BMMSCs (Figures 1(f)–1(h)). Additionally, relative expression levels of *ALP* and *Runx2* also showed an upward trend after KGN induction (Figures 1(i) and 1(j)). These data indicate that KGN could enhance BMMSC osteogenic differentiation without affecting cell proliferation activity.

### 3.2. KGN Upregulates Autophagic Activity of BMMSCs

**Figure 2:**
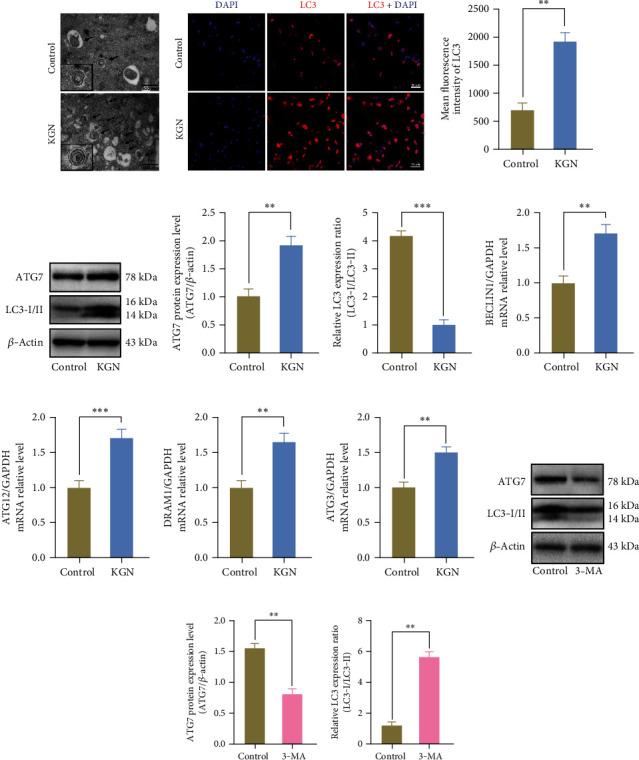
Autophagy is involved in the process of KGN-induced BMMSC osteogenesis. (a) The ultrastructural features of autophagic vacuoles by TEM. Black arrows suggest autophagosomes or autophagolysosomes in BMMSCs. Scale bar = 500 nm. Detection of LC3 (red) and DAPI (blue) by immunofluorescent staining (b) in the control and KGN-treated BMMSCs and semiquantification analysis (c). KGN upregulated the expression of autophagic marker LC3. Scale bar = 50 *μ*m. ^∗∗^*p* < 0.01, independent Student's *t* test. (d) The effects of KGN on autophagy-associated markers were evaluated by western blotting, (e, f) semiquantification of (d), and qRT-PCR of autophagy markers (g–j). KGN promoted the expression of ATG7 and downregulated the LC3-I/LC3-II ratio. ^∗∗^*p* < 0.01 and ^∗∗∗^*p* < 0.001, independent Student's *t* test. (k) The effects of inhibitor 3-MA on autophagy-associated markers were evaluated by western blotting. (l, m) Semiquantification of (k). 3-MA inhibited the expression of ATG7 and upregulated the LC3-I/LC3-II ratio. ^∗∗^*p* < 0.01, independent Student's *t* test.

To evaluate whether KGN affected the autophagic activity of BMMSC during osteogenic differentiation, TEM was conducted to determine the formation of autophagosomes in BMMSCs morphologically, which is considered as the standard for detecting autophagy [34] (Figure 2(a)). LC3, as a marker of autophagy, was also analyzed by immunofluorescence staining. The results suggested that the expression level of LC3 increased distinctly after KGN induction (Figures 2(b) and 2(c)). In addition, a panel of autophagic markers was used to detect autophagic activity. ATG7 and cleaved *LC3* product, LC3II, all presented an elevated level after KGN treatment, proving that KGN is closely associated with autophagy (Figures 2(d)–2(f)). Consistently, results from qRT-PCR also revealed a remarkable increase in the expression of the autophagy-related markers of *Beclin1*, *Atg12*, *Dram1*, and *Atg3* (Figures 2(g)–2(j)). Taken together, these results suggest reinforcement of autophagic activity in KGN-treated BMMSCs. To confirm this result, an early autophagy inhibitor 3-MA was used and effectively suppressed autophagic level in BMMSCs, as assessed by western blotting (Figures 2(k)–2(m)).

### 3.3. KGN Improves Osteogenesis of BMMSCs through Autophagy Activation

**Figure 3:**
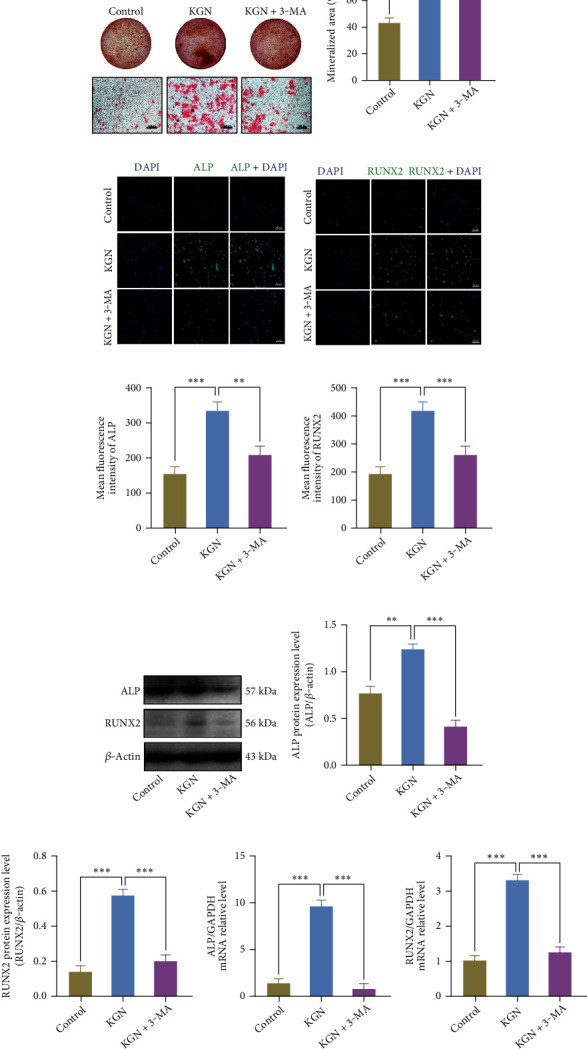
KGN increases the osteogenic capability of BMMSCs via upregulating autophagy. ARS (a) and semiquantification (b) of mineralized nodules. 3-MA-mediated autophagy suppression inhibited KGN-induced BMMSC osteogenesis. Scale bar = 500 *μ*m. ^∗^*p* < 0.05 and ^∗∗∗^*p* < 0.001, one-way ANOVA test. (c, d) Detection of ALP (green), Runx2 (green), and DAPI (blue) by immunofluorescent staining in BMMSCs. (e, f) Semiquantification of (c) and (d). 3-MA-mediated autophagy suppression inhibited KGN-induced expression of osteogenic genes. Scale bar = 50 *μ*m. ^∗∗^*p* < 0.01 and ^∗∗∗^*p* < 0.001, one-way ANOVA test. (g) Western blotting, (h, i) semiquantification of (g), and qRT-PCR assay (j, k) of ALP and Runx2. 3-MA-mediated autophagy suppression inhibited KGN-induced expression of osteogenic genes. ^∗∗^*p* < 0.01 and ^∗∗∗^*p* < 0.001, one-way ANOVA test.

Autophagy plays an important role during BMMSC differentiation into primary functional cells in bone formation, regulating their physiological functions to maintain bone homeostasis [35]. Although KGN has been shown to elevate autophagic activity in BMMSCs and promote osteogenic differentiation, the underlying mechanism remains elusive. Therefore, 3-MA was applied prior to the induction by KGN to identify its autophagic role during BMMSC osteogenic differentiation. On day 14 after treatment, 3-MA was found to have attenuated the number of calcium nodules induced by KGN (Figures 3(a) and 3(b)). Compared with the control group, immunofluorescence staining also indicated that the KGN-induced expression of both ALP and Runx2 was suppressed by 3-MA (Figures 3(c)–3(f)). Consistent with this, the protein and mRNA levels of ALP and Runx2 showed an appreciable decline when 3-MA was involved (Figures 3(g)–3(k)). This suggested autophagy performs important functions in osteogenic differentiation caused by KGN, and suppression of autophagy leads to the inhibition of osteogenic differentiation.

### 3.4. KGN Promotes Osteogenesis of BMMSCs through Smad1/5/9 Signal after Autophagy Activation

**Figure 4:**
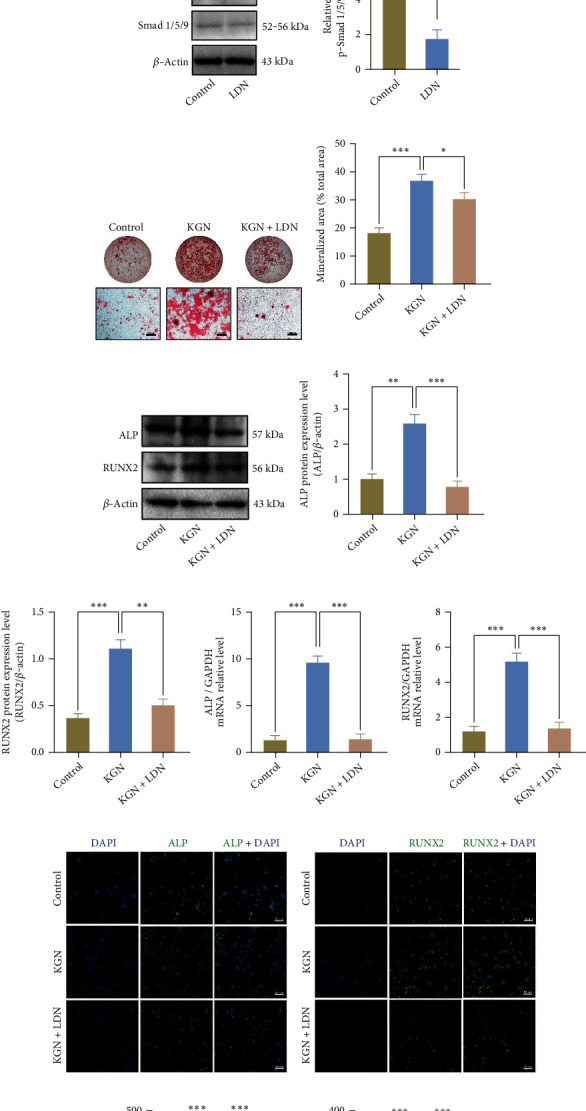
KGN elevates autophagic activities in BMMSCs and prompts osteogenesis through Smad1/5/9 signal pathway. Western blotting (a) and semiquantification (b) of Smad and phosphorylation level of Smad1/5/9. 3-MA-mediated autophagy suppression inhibited KGN-induced Smad1/5/9 phosphorylation increase. ^∗∗∗^*p* < 0.001, one-way ANOVA test. Western blotting (c) and semiquantification (d) of the phosphorylation level of Smad1/5/9 in the control and LDN-treated BMMSCs. LDN effectively inhibited phosphorylation level of Smad 1/5/9. ^∗∗^*p* < 0.01, independent Student's *t* test. ARS detection (e) and semiquantification (f) of mineralized nodules. LDN inhibited KGN-induced osteogenesis. Scale bar = 500 *μ*m. ^∗^*p* < 0.05 and ^∗∗∗^*p* < 0.001, one-way ANOVA test. (g) Western blotting, (h, i) semiquantification of (g), and (j, k) qRT-PCR assay of ALP and Runx2. LDN inhibited KGN-induced expression of osteogenic genes. ^∗∗^*p* < 0.01 and ^∗∗∗^*p* < 0.001, one-way ANOVA test. (l, m) Detection of ALP (green), Runx2 (green), and DAPI (blue) by immunofluorescent staining in BMMSCs. (n, o) Semiquantification of (l) and (m). LDN inhibited KGN-induced expression of osteogenic genes. Scale bar = 50 *μ*m. ^∗∗∗^*p* < 0.001, one-way ANOVA test.

Considering the crucial role of Smad signaling in MSC osteogenic differentiation [12], we investigated whether the Smad1/5/9 pathway participates in KGN-induced osteogenic differentiation. To test whether autophagy and the Smad1/5/9 pathways mediated osteogenic differentiation, 3-MA and LDN were applied for the above two inhibitions, respectively. As assessed by western blotting, KGN upregulated the phosphorylation of Smad1/5/9, and 3-MA attenuated this effect (Figures 4(a) and 4(b)), whereas Smad1/5/9 remained stable. Moreover, the phosphorylation level of Smad1/5/9 was compressed by LDN (Figures 4(c) and 4(d)). After treatment of LDN, the calcium nodules were significantly decreased compared with the KGN group by ARS, suggesting the osteogenesis effect was greatly weakened by LDN (Figures 4(e) and 4(f)). Consistently, LDN also suppressed both the protein and mRNA levels of ALP and Runx2 induced by KGN (Figures 4(g)–4(k)). Also, similar findings were also presented in immunofluorescence staining (Figures 4(l)–4(o)), suggesting LDN blocked the KGN-induced increase in ALP and Runx2 expression levels.

**Figure 5:**
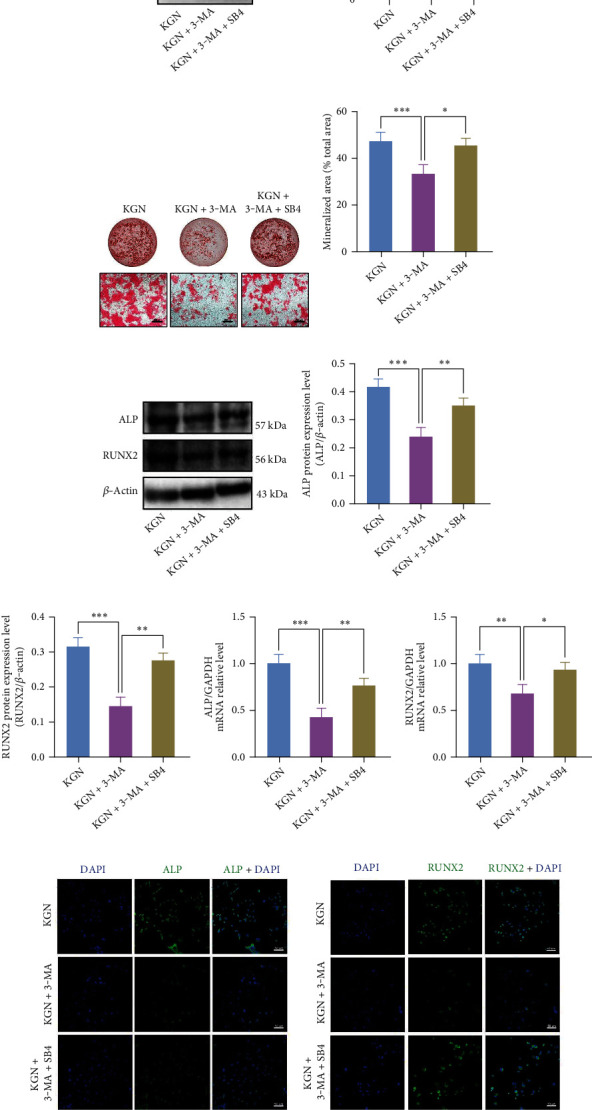
BMMSC osteogenic level promoted by KGN is suppressed by 3-MA, whereas rescued by SB4. Western blotting assay (a) and semiquantification (b) of Smad1/5/9 and phosphorylation level of Smad1/5/9 in the control and SB4-treated BMMSCs. SB4 promoted phosphorylation level of Smad1/5/9. ^∗∗∗^*p* < 0.001, independent Student's *t* test. Western blotting assay (c) and semiquantification (d) on Smad1/5/9 and phosphorylation level of Smad1/5/9. 3-MA suppressed KGN-induced increased phosphorylation level of Smad1/5/9, whereas SB4 reversed this effect. ^∗^*p* < 0.05 and ^∗∗∗^*p* < 0.001, one-way ANOVA test. ARS (e) and semiquantification (f) of mineralized nodules. 3-MA restrained KGN-induced BMMSC osteogenesis, whereas SB4 reversed this effect. Scale bar = 500 *μ*m. ^∗^*p* < 0.05 and ^∗∗∗^*p* < 0.001, one-way ANOVA test. (g) Western blotting, (h, i) semiquantification of (g), and (j, k) qRT-PCR of ALP and Runx2. 3-MA suppressed KGN-induced expression of osteogenic genes whereas SB4 reversed this effect. ^∗^*p* < 0.05, ^∗∗^*p* < 0.01, and ^∗∗∗^*p* < 0.001, one-way ANOVA test. (l, m) Detection of ALP (green), Runx2 (green), and DAPI (blue) by immunofluorescent staining in BMMSCs. (n, o) Semiquantification of (l) and (m). Scale bar = 50 *μ*m. ^∗^*p* < 0.05 and ^∗∗∗^*p* < 0.001, one-way ANOVA test.

Besides, to further confirm the involvement of Smad1/5/9 pathways in KGN-induced osteogenesis, we employed its activator SB4 (Figures 5(a) and 5(b)) after KGN treatment over the course of 24 h. As shown above, 3-MA inhibited the phosphorylation of Smad1/5/9, whereas this effect was rescued by SB4 (Figures 5(c) and 5(d)). Consistent with this, ARS staining suggested that calcium salt deposits of KGN were reduced by 3-MA, whereas SB4 reduced the suppression effect (Figures 5(e) and 5(f)). These results were verified using western blotting (Figures 5(g)–5(i)), qRT-PCR (Figures 5(j) and 5(k)), and immunofluorescence staining (Figures 5(l)–5(o)). These results confirmed that KGN enhanced BMMSC osteogenic differentiation through the Smad1/5/9 pathways after autophagy activation.

## 4. Discussion

As an essential part of bone tissue engineering, BMMSCs have a multilineage differentiation profile, migration and paracrine effects, and low immunogenicity risk, making them widely researched for osteoporosis and bone defect repair [36]. In recent years, studies of KGN have mostly focused on regenerative medicine. Most researches have concentrated on KGN roles for cartilage repair and osteoarthritis treatment. Johnson et al. [3] first demonstrated that KGN could induce MSC chondrogenic differentiation by enhancing the nuclear translocation of CBF*β* in order to develop complexes with RUNX1 as well as facilitating to express COL II. So far, the chondrogenic promotion and protection functions of KGN have also been proved [37, 38]. Wang et al. [7] revealed that KGN attenuated reactive oxygen species in intracellular environments and promoted osteogenic differentiation of human BMMSCs. However, the relationship between KGN and osteogenesis improvement is not yet fully understood. In this study, we confirmed the significant inducing role of KGN in promoting BMMSC osteogenesis by upregulating autophagy activity and the Smad1/5/9 pathway. Several studies have revealed an obvious increase in the expression of early osteogenesis-associated markers, such as ALP and Runx2, during BMMSC osteogenic differentiation [39–41]. In our study, KGN elevated the expression level of ALP and Runx2 dominantly. Meanwhile, the proliferation activity of BMMSCs was not affected, indicating that KGN promoted BMMSC osteogenic differentiation capacity without affecting their proliferation ability.

Autophagy, a homeostatic mechanism under various conditions, is required to eliminate cellular waste produced during the quiescent stage of stem cells [42]. The modulation of autophagy in MSCs could affect their differentiation capacity, as well as the fate of proliferation, activation, and function [42]. Past research has shown that autophagy plays an indispensable part in bone remodeling regulation [21]. In our study, the protein levels of autophagy-related genes of Atg7 as well as LC3-II/LC3-I ratio were both elevated after KGN treatment, and the expressions of *Beclin1*, *Atg12*, *Dram1*, and *Atg3* were enhanced likewise. These results reflect the role of KGN in promoting autophagic activities of BMMSCs. Additionally, 3-MA, an autophagy inhibitor, was used to confirm the involvement of autophagy during KGN treatment. As a result of 3-MA-induced autophagy suppression, the protein and mRNA levels of ALP and Runx2 in KGN-treated BMMSCs were sharply downregulated, and osteogenic differentiation activity was attenuated. The finding demonstrated that autophagy is essential in KGN-induced osteogenic differentiation.

Studies have shown that autophagy is involved in TGF-*β*/Smad pathway [14, 43]. Zhang et al. demonstrated that insulin inhibits the osteogenic level of BMMSCs by suppressing autophagic activities and enhancing premature senescence via the TGF-*β*_1_ signaling pathway [44]. The phosphorylation of Smad2/3 is also affected by autophagy, which affects a range of functions [45], such as epithelial-to-mesenchymal transition and cell migration [46]. However, the impact of autophagy on Smad1/5/9 protein remains unclear, and current studies on the correlation between autophagy and Smad proteins mostly focus on the functions of cells such as the fibroblasts and endothelial cells. In this study, KGN was found to induce phosphorylation level of Smad1/5/9 in BMMSCs. A study by Decker et al. also proposed that KGN could prompt mouse embryo limb development mainly by upregulating the phosphorylation of Smad2/3 of E11.5 limb bud mesenchymal cells [10]. This phenomenon might be attributed to stemness properties and degrees of differentiation, and the effects of KGN on various Smad phosphorylation levels in embryonic or adult stem cells could impact the osteogenic capacity. Additionally, our results further suggested that LDN [47], a selective inhibitor of the Smad signaling pathway, could suppress the differentiation ability of BMMSCs, whereas the activator SB4 [48] could rescue the inhibiting impacts of 3-MA on the osteogenic differentiation in BMMSCs. These results indicate that KGN induced autophagic activity of BMMSCs and enhanced osteogenic differentiation ability by upregulating the phosphorylation level of Smad1/5/9, which was not found in previous studies. Therefore, our findings suggest that autophagy and Smad1/5/9 play important roles not only in fibroblasts or endothelial cells but also in BMMSCs for osteogenic differentiation. However, although the roles of KGN in BMMSC osteogenic differentiation have been shown here in vitro, more in vivo studies should be further conducted. Therefore, research on the specific functions and mechanisms of KGN would be indispensable, for example, the effects of KGN on bone repair and reconstruction in different cell niches (different age stages, various types of bone defects, osteoporosis model, etc.) [49, 50], and the relationship between KGN and osteogenesis/chondrogenesis under different conditions should also be elucidated. In addition, the application of KGN in vivo is worthy of further exploration, such as combining KGN with biological scaffold materials, seed cells and growth factors to meet the needs of bone tissue engineering, and even suitable drug delivery systems (DDS) to facilitate controlled delivery of KGN [51–53]. More in-depth research to identify the mechanisms of KGN including autophagy-related gene knockout and Smad pathway validation in vivo is also required so as to better understand the role of KGN in bone regeneration.

## 5. Conclusions

**Figure 6:**
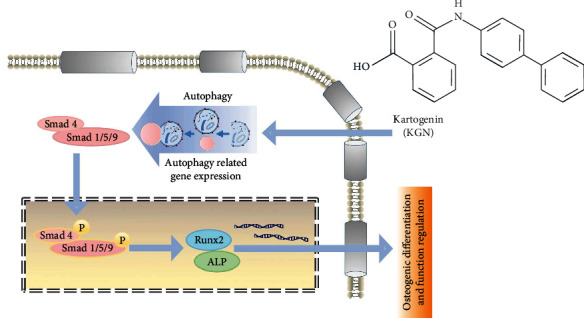
Schematic diagram of the study. KGN promotes autophagic activity of BMMSCs and upregulates the phosphorylation level of Smad1/5/9, thereby enhancing osteogenesis of BMMSCs.

In summary, our study has revealed that KGN plays a key role in promoting osteogenic differentiation of BMMSCs via the modulation of autophagy and phosphorylation level of Smad1/5/9 (Figure 6). The research also improves the understanding of the properties of MSCs and the roles of Smad isoforms as well as the autophagy/Smad1/5/9 signaling pathway, thereby facilitating further studies to explore osteogenic capacity of MSCs in bone regeneration and clinical treatment of bone tissue engineering.

## Figures and Tables

**Table 1:** Primer sequences for qRT-PCR.

Gene name	Primer sequence
*Runx2-F*	CCGGGAACCAAGAAGGCACA
*Runx2-R*	AGGCGGGACACCTACTCTCA
*Alp-F*	GTGACTACCACTCGGGTGAAC
*Alp-R*	CTCTGGTGGCATCTCGTTATC
*Beclin1-F*	AGGCGAAACCAGGAGAGAC
*Beclin1-R*	CCTCCCCGATCAGAGTGAA
*Atg12-F*	TCCCCGGAACGAGGAACTC
*Atg12-R*	TTCGCTCCACAGCCCATTTC
*Dram1-F*	TCATCTCCTACGTGGTCGC
*Dram1-R*	CTGCGCCAAGAAATGCAGAG
*Atg3-F*	ACACGGTGAAGGGAAAGGC
*Atg3-R*	TGGTGGACTAAGTGATCTCCAG
*GAPDH-F*	TGGCCTTCCGTGTTCCTAC
*GAPDH-R*	GAGTTGCTGTTGAAGTCGCA

## Data Availability

The authors confirm that all data underlying the findings are fully available. Further inquiries can be directed to the corresponding authors.

## Supplementary Materials

Supplementary Materials Supplementary Figure 1: the titration data of KGN for BMMSC osteogenesis. (A) ARS and (B) semiquantification of mineralized nodules of KGN-induced BMMSC osteogenesis at different concentrations from 0.1 *μ*M to 100 *μ*M. Scale bar = 500 *μ*m. (C, D) qRT-PCR showed the expression levels of ALP and Runx2 at different concentrations of KGN. ^∗^*p* < 0.05 and ^∗∗∗^*p* < 0.001, one-way ANOVA test.
