# Supplementary material for: Kartogenin Improves Osteogenesis of Bone Marrow Mesenchymal Stem Cells via Autophagy
Source: Stem Cells Int. 2022 Dec 22;2022:1278921. doi: 10.1155/2022/1278921 (PMC9800103; doi:10.1155/2022/1278921)
Supplement: Supplementary Materials — Supplementary Figure 1: the titration data of KGN for BMMSC osteogenesis. (A) ARS and (B) semiquantification of mineralized nodules of KGN-induced BMMSC osteogenesis at different concentrations from 0.1 μM to 100 μM. Scale bar = 500 μm. (C, D) qRT-PCR showed the expression levels of ALP and Runx2 at different concentrations of KGN. ∗p < 0.05 and ∗∗∗p < 0.001, one-way ANOVA test. [file 1278921.f1.docx]

**Article Title: Kartogenin Improves Osteogenesis of Bone Marrow Mesenchymal Stem Cells via Autophagy**

Huichun Yan ^1,2,3^, Tingting Yu ^1,2,3^, Jing Li^1,2,3^, Ting Zhang ^1,2,3^, Qian Li ^1,2,3^, Yanheng Zhou ^1,2,3^* and Dawei Liu^1,2,3,4^*

^1^Department of Orthodontics, Peking University School and Hospital of Stomatology, Beijing 100081, China

^2^National Clinical Research Center for Oral Diseases & National Engineering Laboratory for Digital and Material Technology of Stomatology, Beijing 100081, China

^3^Beijing Key Laboratory of Digital Stomatology, Beijing 100081, China

^4^State Key Laboratory of Military Stomatology, Xi’an 710000, China

^*^Corresponding authors should be addressed to Yanheng Zhou; [yanhengzhou@vip.163.com](mailto:yanhengzhou@vip.163.com) and Dawei Liu; [liudawei@bjmu.edu.cn](mailto:liudawei@bjmu.edu.cn)

HuiChun Yan and Tingting Yu have contributed equally to this work as co-first authors.

**Supplementary Figure**

**
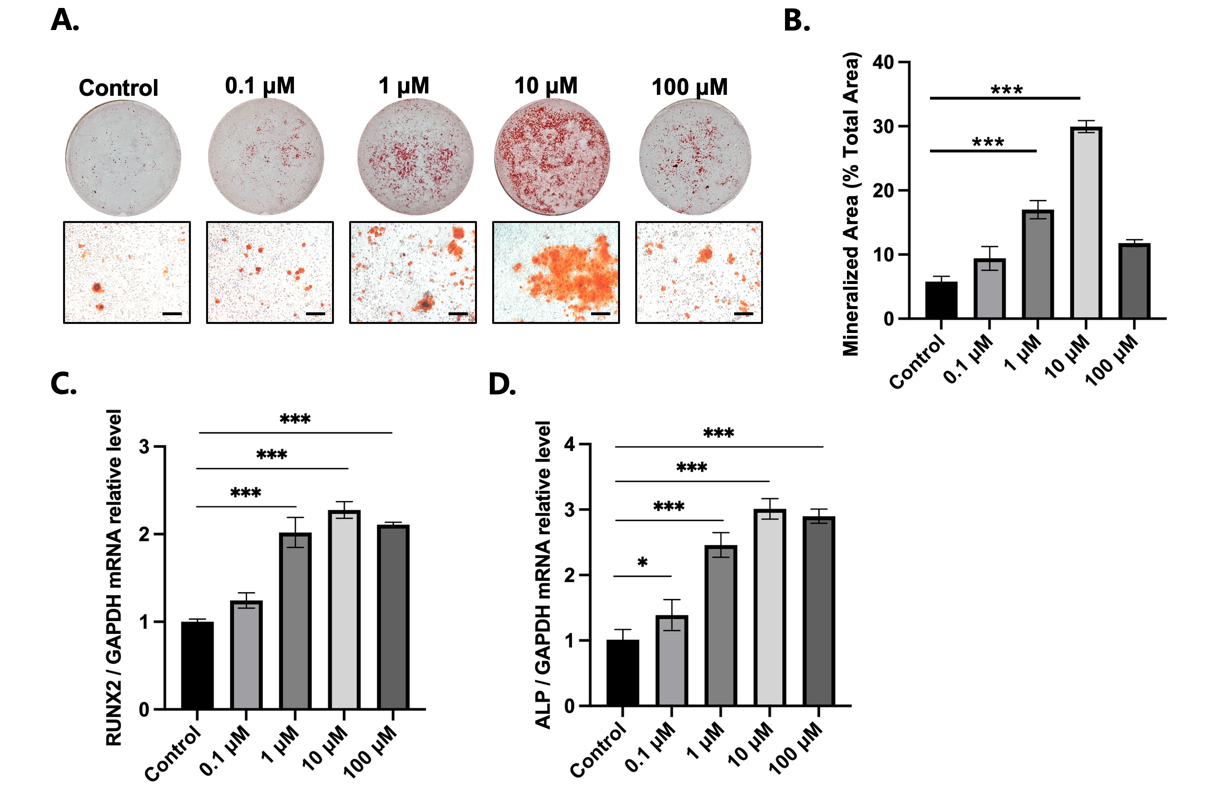
**

**Supplementary Figure 1: The titration data of KGN for BMMSC osteogenesis.** (A) ARS and (B) semi-quantification of mineralized nodules of KGN-induced BMMSC osteogenesis at different concentrations from 0.1μM to 100 μM. Scale bar = 500 μm. (C-D) qRT-PCR showed the expression levels of RUNX2 and ALP at different concentrations of KGN. **p* < 0.05, ****p* < 0.001, one-way ANOVA test.
